# Enhancing Stability of Reprocessed Acrylonitrile–Butadiene–Styrene (ABS) Polymers from Sorted WEEE Streams for Filament Extrusion

**DOI:** 10.3390/polym18080971

**Published:** 2026-04-16

**Authors:** Christina Podara, Christos Panagiotopoulos, Dionysia Kouranou, Christos Tsirogiannis, Melpo Karamitrou, Tatjana Kosanovic Milickovic, Stamatina Vouyiouka, Costas Charitidis

**Affiliations:** 1 Research Lab of Advanced, Composite, Nano Materials & Nanotechnology (R-NanoLab), School of Chemical Engineering, National Technical University of Athens, 9 Heroon, Polytechniou St., Zografos, 157 80 Athens, Greece; cpodara@chemeng.ntua.gr (C.P.); christsiro@chemeng.ntua.gr (C.T.); mkaramitru@chemeng.ntua.gr (M.K.); tkosanovic@chemeng.ntua.com (T.K.M.); 2 Laboratory of Polymer Technology, School of Chemical Engineering, National Technical University of Athens, 9 Heroon, Polytechniou St., Zografos, 157 80 Athens, Greece; chpanagiotopoulos@mail.ntua.gr (C.P.); dkouran@gmail.com (D.K.); mvuyiuka@central.ntua.gr (S.V.)

**Keywords:** WEEE plastics, styrenic polymers, mechanical recycling, antioxidant stabilization, filament extrusion

## Abstract

The mechanical recycling of styrenic polymers from waste electrical and electronic equipment (WEEE) is often limited by thermomechanical degradation occurring during repeated processing. In this work, the degradation behaviour of acrylonitrile–butadiene–styrene (ABS) recovered from sorted WEEE streams was systematically investigated through multiple extrusion cycles, and the effectiveness of antioxidant stabilization was evaluated. Progressive degradation was assessed by chemical structure, rheological, thermal and mechanical testing, and colorimetric analysis. Repeated processing resulted in increased melt flow, altered viscoelastic behaviour, molecular weight reduction, deterioration of mechanical properties, and discoloration. To mitigate these effects, antioxidant-stabilized compounds were prepared and subjected to identical reprocessing pathways. The addition of antioxidants effectively reduced chain scission, stabilized rheological properties, and limited colour changes during reprocessing. Furthermore, the processability of the optimized recycled ABS is demonstrated through filament extrusion for fused filament fabrication, highlighting its potential for high-value additive manufacturing applications. These results demonstrate that appropriate stabilization strategies can significantly enhance the process stability and recyclability of styrenic polymers from WEEE streams, supporting their use in higher-value applications within a circular economy framework.

## 1. Introduction

The rapid growth of electrical and electronic equipment has led to a significant increase in waste electrical and electronic equipment (WEEE), which represents one of the fastest-growing waste streams worldwide. The plastics cover a substantial fraction of the total EEE waste, around 25–30% [1], with styrenic polymers such as acrylonitrile–butadiene–styrene (ABS) and high-impact polystyrene (HIPS) being among the most used materials [2]. More specifically, WEEE typically consists of around 80 wt% styrenics (HIPS and ABS), 10 wt% polypropylene (PP), and 10% others [3]. The recycling of these plastics is of major importance for resource conservation and reduction in environmental impact. A study conducted by Campolina et al. [1] reports that, comparing the mechanical recycling of ABS and HIPS from WEEE to the virgin production, it can cut energy use by ~90% and CO_2_ emissions into the atmosphere by 84% in the case of HIPS and 87% by recycling ABS. By using the LCA tool, this study helped large-scale WEEE plastic recycling in China [4] and Korea [5] to yield substantial GHG savings compared with virgin plastics, though ozone-depleting refrigerant leakage remains an issue at the system level.

However, the effective mechanical recycling of WEEE-derived plastics remains challenging. The compositional heterogeneity, multiple thermal histories, and the cross-contamination with legacy additives of ABS streams pose a great intrinsic challenge. WEEE ABS is typically a mixture of multiple grades, with variable acrylonitrile/butadiene/styrene ratios and mineral fillers such as CaCO_3_, silica, talc, TiO_2_, ~9 wt% [6,7,8]. Also, mixed styrenic streams (HIPS/ABS/PS) are usually contaminated with other polymers such as PP, which sharply reduces tensile and especially impact strength even in small concentrations such as 2–4% [9]. This can be tackled with the incorporation of small amounts of compatibilizers like SEBS or SBR, which turn styrenic polymers from brittle to ductile, enabling high impact and tensile properties, comparable to virgin or conventional polymers [3,6,10,11].

Furthermore, styrenic WEEE plastics often contain brominated flame retardants (BFRs); Charitopoulou et al. found that 41% of the samples (7 out of 17) contained bromine. The bromine content varied among the samples of each category and among the different categories of devices [12]; in many cases, the BFR content is above regulatory limits [13,14]. BFRs complicate mechanical and chemical recycling and can form hazardous emissions if incinerated or pyrolyzed without pretreatment [14,15]. Debromination/extraction is technically feasible and can yield > 94% BFR removal, leaving a residual content of less than 50 ppm, with limited loss of bulk ABS properties, but often reduces impact toughness and has a high energy/solvent footprint [16,17,18,19]. Furthermore, advanced sorting techniques can eliminate the presence of BFRs, so only bromine-free ABS enters mechanical recycling. Fast BR screening is enabled by XRF and NIR combinations [20], while industrial NIR systems can sort ABS/HIPS/ABS-PC with >90% purity and high efficiency, forming high-quality styrenic recyclate when applied after an initial BFR screening [21]. Moreover, utilization of Raman spectroscopy coupled with machine-learning algorithms and NIR-based auto-sorting further improves correct identification of ABS, ABS/PC, and PS, including black plastics, with purities up to ~80–99% [22,23].

Another barrier in the mechanical recycling of these streams is related to ageing phenomena such as thermo-oxidative degradation and UV exposure, which can significantly alter the molecular structure of ABS prior to recycling, leading to variability in properties and processing behaviour. Natural photo-ageing and UV exposure preferentially degrade the butadiene rubber phase, leading to fragmentation, smaller particle size, and embrittlement; ABS and recycled ABS show the greatest particle size reduction among e-plastics [24]. UV ageing increases hydrophilicity and decreases crystallinity, which can also change additive release behaviour [25]. Teixeira et al. showed that repeated extrusion/injection cycles (up to 24 cycles) cause moderate thermo-oxidative degradation: MFI rises 11–15%, impact strength of WEEE ABS drops about ~14% after a few cycles, while tensile/flexural properties and T_onset_ (~381–386 °C) remain almost unchanged [26].

More specifically, repeated melt processing exposes ABS to combined thermal and shear stresses, which promote degradation reactions in its two phases: polybutadiene (PB) phase and the styrene–acrylonitrile (SAN) matrix. Regarding the PB phase, the oxidation starts on PB’s double bonds, forming hydroperoxides that decompose into radicals, causing PB chain scission as well as crosslinking [27,28,29]. That leads to higher moduli and stiffer surfaces, as well as viscosity raising [29,30]. Regarding the SAN phase degradation, it occurs at higher temperatures or longer times; the thermo-oxidation causes backbone chain scission, lowering molar mass and causing severe embrittlement and impact strength drop [30,31]. Last but not least, reprocessing and thermo-oxidative ageing particularly enhance yellowing, even when bulk mechanical properties change modestly [32], due to conjugated chromophores that are formed (conjugated carbonyls and vinyl/aromatic structures).

From the processability perspective, as already mentioned, the heterogeneous WEEE ABS grades differ in composition, thermal history, additivation, and BFR content, complicating melt blending and filament production; some grades need compatibilizers or solvent pretreatments for stable extrusion [18,33]. Although many studies show that ABS from WEEE can still reach near-virgin tensile properties, the impact strength and elongation at break are the first properties to fail, especially after ageing, PP contamination, or BFR extraction [26,34,35].

To mitigate these effects, the use of antioxidant stabilizers is a widely adopted strategy in polymer processing and recycling. Primary and secondary antioxidants act by scavenging free radicals and decomposing hydroperoxides formed during thermo-oxidative degradation, thereby limiting chain scission and preserving molecular integrity. More specifically, primary (phenolic) antioxidants scavenge macroradicals (R·, ROO·), interrupting the autoxidation chain, while secondary (phosphite) antioxidants decompose hydroperoxides (ROOH) to non-radical products [36]. In the context of mechanical recycling, antioxidant addition has been shown to improve property retention and extend the processing lifetime of recycled polymers [37]. However, the efficiency of stabilization strongly depends on the initial state of degradation and the complexity of the recycled feedstock [38].

While previous studies have investigated the stabilization of virgin or model ABS systems during reprocessing, significantly fewer works have focused on real post-consumer WEEE-derived materials, which inherently combine prior ageing, compositional heterogeneity, and contamination. Moreover, most existing studies address degradation through isolated characterization techniques, often lacking a comprehensive correlation between molecular, rheological, mechanical, and optical property evolution during repeated processing.

In this context, the present work distinguishes itself by (i) employing industrially relevant ABS recovered from sorted WEEE streams, (ii) providing a systematic multi-technique assessment of degradation through chemical, rheological, thermal, mechanical, and colorimetric analysis, and (iii) demonstrating the practical valorisation of the optimized material via filament extrusion for fused filament fabrication. This combined approach enables a more realistic evaluation of recyclability and end-use potential compared to conventional studies based on virgin or laboratory-grade materials.

More specifically, the aim of this study is to systematically investigate the thermomechanical degradation behaviour of real post-consumer ABS recovered from sorted WEEE streams and to evaluate the effectiveness of antioxidant stabilization under multiple reprocessing cycles. The evolution of rheological, chemical, thermal, mechanical, and colour properties is assessed to quantify degradation and stabilization effects, to enhance the ability of designing robust recycling strategies for high-quality reuse of WEEE. Finally, the processability of the optimized recycled ABS is demonstrated through filament extrusion for fused filament fabrication, highlighting its potential for high-value additive manufacturing applications.

## 2. Materials and Methods

### 2.1. Materials

A real post-consumer polymer waste stream coming from refrigerators was kindly provided by Coolrec Plastics Plant (Waalwijk, The Netherlands). The material had been sorted in the Coolrec sorting lines, yielding high purity to one polymer-rich stream (~97% for ABS, purity provided by Coolrec Plastics). For the stabilization, two different formulations with readily available additives were examined. Specifically, both formulations include a primary and a secondary antioxidant, (a) Irganox^®^ 1076 and Irgafos^®^ 168, as well as (b) Irganox^®^ 245 and Irgafos^®^ 168, respectively, purchased by BASF Hellas S.A., Athens, Greece. Tetrahydrofuran (THF) (HPLC grade, ≥99.8%, CARLO ERBA Reagents S.A.S, Val-de-Reuil, France) was used as received without further purification.

### 2.2. Processes

**Figure 1:**
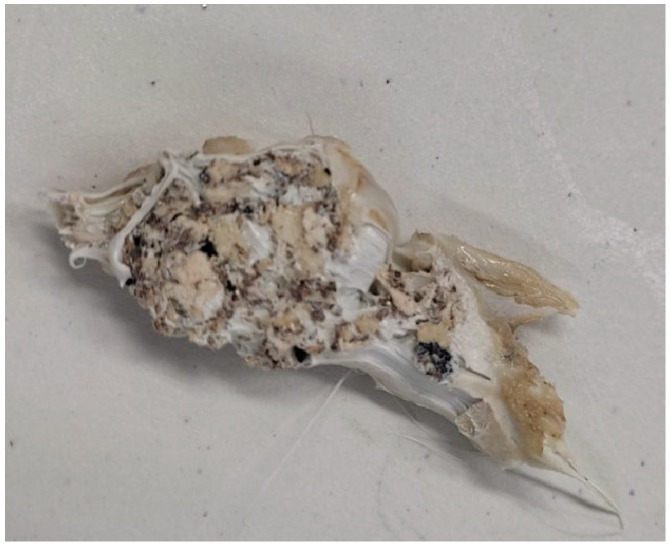
Impurities on the melt filter screen.

Initially, the ABS material was melt-filtrated in a laboratory-scale co-rotating twin-screw extruder (Process 11, Thermo Fisher Scientific, Karlsruhe, Germany), equipped with a lab-scale melt filter screen of mesh size 100 (149 microns), to remove some of the impurities. As a 100% post-consumer EEE waste suffering from heterogeneity and some contamination, melt filtration and extrusion are necessary [39]. The extrusion temperature profile was set within the range of 200–220 °C. Figure 1 shows the filter screen after processing 100 g of ABS material. The melt filtration certainly improved the material’s purity.

Afterwards, a degradation study by multiple extrusion cycles was conducted on ABS using a single-screw extruder (Plasti-Corder PLE330, Brabender, Duisburg, Germany). The material was subjected to five extrusion cycles at a temperature of 250 °C, screw speed of 50 rpm, approaching closer to real industrial processing conditions. Finally, the extrudate was cooled in a water bath. The processing temperature of 250 °C was selected as it lies within the typical industrial processing window for ABS (220–260 °C), ensuring representative industrial processing conditions.

Both stabilization systems were attempted by incorporating a 50:50% wt. mixture of a primary (Irganox^®^ 1076 or Irganox^®^ 245) and secondary antioxidant (Irgafos^®^ 168). Masterbatches containing 10 wt.% total antioxidant content were first prepared to ensure homogeneous dispersion of the stabilizers within the ABS matrix. Two formulations were produced: (a) 5.0 wt.% Irganox^®^ 1076 combined with 5.0 wt.% Irgafos^®^ 168, and (b) 5.0 wt.% Irganox^®^ 245 combined with 5.0 wt.% Irgafos^®^ 168. Prior to compounding, the ABS was ground to facilitate uniform premixing with the antioxidant additives, which were in powder form. This step was considered essential to enhance the distribution of the stabilizers during subsequent melt processing. The masterbatches were then prepared using a co-rotating twin-screw extruder (Process 11, Thermo Fisher Scientific, Karlsruhe, Germany) at a melt temperature of 210 °C and a screw speed of 80 rpm, and pelletised. Masterbatch pellets, well mixed with ABS pellets, were used to produce a compound of ABS with a final concentration of 0.5 wt.% of each antioxidant. Compounding was performed in a bigger scale twin-screw extruder (Collin ZK 25 × 24D, Dr. Collin GmbH, Ebersberg, Germany) at 210 °C and 130 rpm. Process parameters are presented in Table 1.

The two restabilized materials were then reprocessed at 250 °C, respectively, for five cycles, in the single-screw extruder under the same conditions as the neat material. All extrudates from all processes were characterized similarly. All prepared sample formulations and their respective nomenclatures are summarized in Table 2.

### 2.3. Preparation of ABS Filaments

Filament extrusion was aimed at producing a uniform filament with a nominal diameter of 1.75 ± 0.05 mm, suitable for fused filament fabrication (FFF). A melt pump and a laser-based diameter monitoring system were used to improve dimensional stability. The extruded filament was cooled in a water bath and subsequently wound onto spools.

### 2.4. Characterizations

#### 2.4.1. Melt Flow Rate (MFR)

MFR (g/10 min) was measured at 200 °C and 5 kg, according to ISO 1133 standard [40], using a Dynisco model 4000 capillary rheometer (Dynisco LLC, Franklin, MA, USA). Measurements were performed in triplicate, and MFR was expressed in grams of polymer flowing per 10 min (Equation (1)):

(1) $$MFR\;\left(200\;{}^{\circ}C,\;5\;kg\right)\;=\;\frac{600}{t}m_{avg}$$

where t (s) is the time between consecutive cuts of the molten material (cut-off interval) and m_avg_ (g) is the average mass of the material extruded.

#### 2.4.2. Parallel Plate Rheology

The dynamic rheological behaviour of ABS was analyzed on a HAAKE MARS 40 (Thermo Fisher Scientific Inc., Waltham, MA, USA) rheometer using a parallel plate configuration with a 25 mm diameter. The storage modulus (G′), loss modulus (G″) and complex viscosity (η*) were monitored as a function of the angular frequency ω in the 0.1–600 rad/s range at T = 220 °C. A strain sweep was first performed to determine the linear viscoelastic limit, and a strain amplitude safely within the linear viscoelastic regime was selected for the dynamic frequency sweep measurements. Measurements were performed in triplicate.

#### 2.4.3. Gel Permeation Chromatography (GPC)

GPC analysis was used for the determination of the average molecular weights and molecular weight distribution of ABS samples. Experiments were performed with the use of an Agilent 1260 Infinity II (Agilent Technologies Inc., Santa Clara, CA, USA) instrument, equipped with a guard column (PL gel 5 μm) and two PL gel MIXED-D 5 μm columns (300 × 7.5 mm). Samples were dissolved in THF at a 2 mg/mL concentration. Elution was carried out with THF at a flow rate of 1 mL/min, and an Agilent 1260 Infinity II refractive index detector (RID) 231 (G7162A) was utilized. The calibration of the instrument was carried out with a polystyrene standard solution of molecular weights ranging from 162 to 500,000 g/mol ( EasiVial^TM^ PS-M vials, Agilent Technologies, Santa Clara, CA, USA). Single measurements were performed.

#### 2.4.4. Differential Scanning Calorimetry (DSC)

DSC measurements were performed in a 1 STARe DSC System (Mettler-Toledo, Greifensee, Switzerland)., through a heating-cooling-heating cycle from 30 to 210 °C under N_2_ flow (20 mL/min) using a rate of 10 °C/min. The glass transition temperature (T_g_, °C) was derived from the 2nd heating cycle. Measurements were performed in duplicate.

#### 2.4.5. Thermogravimetric Analysis (TGA)

TGA was conducted in a Mettler TGA/DSC 1 thermobalance (Mettler-Toledo International Inc., Columbus, OH, USA) with a heating rate of 10 °C/min from 30 to 600 °C under nitrogen flow (10 mL/min). The onset decomposition temperature was defined as the temperature at 5% mass loss (T_d,5%_, °C), the degradation temperature (T_d_, °C) was determined at the maximum rate of mass loss, and the char yield (W, %) was the residue at 600 °C. Approximately 20–30 mg of each sample in the form of pellets were prepared, and measurements were performed in duplicate.

#### 2.4.6. Attenuated Total Reflectance Fourier Transform Infrared Spectroscopy (ATR-FTIR)

ATR-FTIR measurements were performed in an Alpha II (Alpha II, Bruker, Germany) using the Attenuated total reflectance (ATR) method with a diamond crystal. Samples were in the form of films (80–100 μm thickness), prepared in a heated plate hydraulic press operating at 180 °C for 1 min under 50 bar pressure, and four random spectra were collected from each of five films, for a total of twenty individual spectra. A thin film was placed on the aperture, and 32 scans were performed over the spectral range of 400–4000 cm^−1^ at 4 cm^−1^ resolution and averaged across the spectral range to improve the signal-to-noise ratio. Also, OPUS Software^®^ (version 8.5) delivers atmospheric compensation for CO_2_ (in the 2282–2399 cm^−1^ region), and H_2_O (in the 1300–1400 cm^−1^ and 3667–3996 cm^−1^ regions), and the presented ATR-FTIR graphs are the average of the twenty spectra collected.

Degradation was monitored through changes in the relevant spectra along with quantified increases in the carbonyl (1680–1850 cm^−1^) and hydroxyl groups (3200–3650 cm^−1^), as well the reduction in the double bond groups from the PB phase present in ABS, corresponding to the 965 cm^−1^ peak of trans-1,4 (930–990 cm^−1^) and 908 cm^−1^ peak of vinyl-1,2 (890–930 cm^−1^) groups. For a semi-quantitative evaluation of the ATR-FTIR spectra, an index for each of the aforementioned groups was calculated based on the following Equations (2) to (5), by integrating the respective areas and normalizing with the styrenic peak of the benzene ring located at 1494 cm^−1^ (integration region at 475–1510 cm^−1^), which is not expected to degrade.

(2) $$Carbonyl\;group\;index,\;CI=\frac{A_{1620-1780\;{cm}^{-1}}}{A_{1475-1510\;{cm}^{-1}}}$$

(3) $$Hydroxyl\;group\;index,\;HI=\frac{A_{3100-3600\;{cm}^{-1}}}{A_{1475-1510\;{cm}^{-1}}}$$

(4) $$Trans-1,4\;index,\;TI=\frac{A_{930-990\;{cm}^{-1}}}{A_{1475-1510\;{cm}^{-1}}}$$

(5) $$Vinyl-1,2\;index,\;VI\;=\;\frac{A_{890-930\;{cm}^{-1}}}{A_{1475-1510\;{cm}^{-1}}}$$

#### 2.4.7. Mechanical Properties–Uniaxial Tensile Testing

Uniaxial tensile testing was conducted using an Instron 4466 universal testing machine equipped with a 10 kN load cell (Instron Co., Norwood, MA, USA) according to ISO 527-2 [41]. The specimens used were Type 1BA (prepared using an in-house injection moulding), and before testing, all specimens were conditioned according to ISO 291 [42], ensuring uniform temperature and humidity conditions to minimize environmental influences on the results. Testing was carried out at a constant crosshead speed of 5 mm/min. From the obtained stress–strain curves, key tensile properties were evaluated, including yield strength (σ_y_, MPa), modulus of elasticity (E, MPa), and strain at yield (ε_y_, %). For each material, 10 specimens were tested to ensure statistical reliability, and standard deviation (SD) and relative standard deviation (RSD, %) values were calculated to assess the variability of the measurements.

#### 2.4.8. Colour Testing

The Yellowness Index (YI) is a colour change indicator that describes the transition from near-white opaque objects to yellowish ones. The measurements were performed using a calibrated handheld Hach Lange LMG 183 spectrophotometer (Konica Minolta, Tokyo, Japan), directly on the films prepared for ATR-FTIR. YI was determined in terms of XYZ based on the CIE D65 standard luminant or 10° standard observer (ASTM E313-20 [43]), meaning the tristimulus values of colour space (Equation (6)). 10 different points of each film were analyzed, and the average measurement was used.

(6) $$YI(\%)=\frac{C_{X}X-C_{z}Z}{Y}\times 100$$

where X, Y and Z represent the red, green, and blue colour, respectively, and C_x_ and C_z_ are equal to 1.3013 and 1.1498, respectively.

## 3. Results

### 3.1. Stabilized vs. Unstabilised ABS During Reprocessing Cycles

#### 3.1.1. Melt Flow Rate Results

MFR is widely used to screen degradation during recycling because it acts as a reliable and efficient indicator of a polymer’s molecular integrity, hence processing performance. During reprocessing, degradation routes (thermal, mechanical, and oxidative) take place, changing the molecular integrity of the polymeric chains; this is directly linked with the MFR values, either by increasing them due to chain scission, or by reducing them due to branching degradation mechanisms [44].

**Figure 2:**
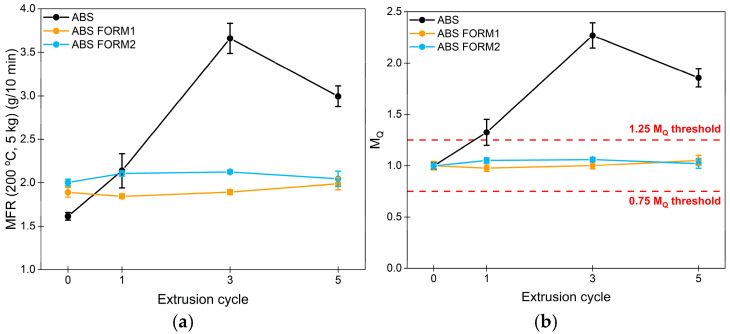
(**a**) MFR (200 °C, 5 kg) and (**b**) M_Q_ of neat ABS, ABS FORM1, and ABS FORM2 as functions of reprocessing cycles.

**Scheme 1:**
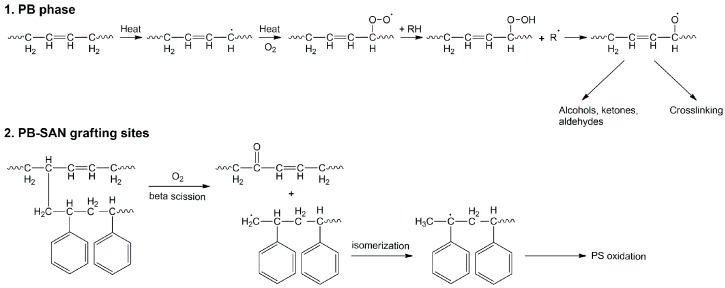
Degradation mechanism of PB and SAN phases of ABS [39].

MFR measurements presented in Figure 2a show that MFR of neat ABS increased up to the third cycle, roughly from 1.61 ± 0.04 g/10 min to 3.66 ± 0.17 g/10 min (127.3% increase), and then decreased again up to the fifth cycle, roughly to 2.99 ± 0.12 g/10 min (85.7% increase). This behaviour was expected as in the case of ABS, where two degradation mechanisms are competing: the dominant one is homolytic bond cleavage (chain scission) due to severe heat and shear stress, which occurs mostly in the styrene-acrylonitrile (SAN) phase; the second degradation mechanism is related with butadiene (PB) phase, in which crosslinking can occur with the recombination of the free radicals that have been formatted by the thermomechanical treatment (Scheme 1) [30]. On the other hand, the stabilized ABS materials better maintained MFR values along reprocessing, roughly from 1.89 ± 0.06 g/10 min to 1.99 ± 0.07 g/10 min for ABS_FORM1 (5.3% increase), and even more for ABS_FORM2, i.e., 2.00 ± 0.04 g/10 min to 2.04 ± 0.09 g/10 min (2.0% increase).

Based on these measurements, the Quality Measure (M_Q_, Equation (7)) was calculated as a ratio of the MFR_i_ of each reprocessing cycle to the initial MFR_0_, as shown in Figure 2b [45], indicating recyclate quality in terms of processability. Thresholds were set in order to assess the prevailing degradation mechanism (chain scission above 1, as well as branching and/or crosslinking below 1), which also helps to define if the recyclate aligns with the guidelines for plastic processability and material performance.

(7) $$\mathrm{Quality}\;\mathrm{Measure}=M_{Q}=\frac{MFR_{i}}{MFR_{0}}\left\{\begin{array}{l} 1\leq \frac{MFR_{i}}{MFR_{0}}\leq 1.25\mathrm{chain}\;\mathrm{scission}\\ 0.75\leq \frac{MFR_{i}}{MFR_{0}}\leq 1\mathrm{branching} \end{array}\right.i=1,2\ldots 5\mathrm{extrusion}\;\mathrm{cycles}$$

As seen in Figure 2b, the quality threshold of M_Q_ = 1.25 was already surpassed from the first extrusion cycle for the unstabilized material and remained above 2 for the whole reprocessing, showcasing that without proper stabilization, ABS rapidly degraded and was unable to be reprocessed. On the contrary, for the stabilised compounds, *M*_Q_ remained within the thresholds for safe reprocessing, meaning that stabilization was effective and prevented degradation up to five extrusion cycles.

#### 3.1.2. Rheological Behaviour

**Figure 3:**
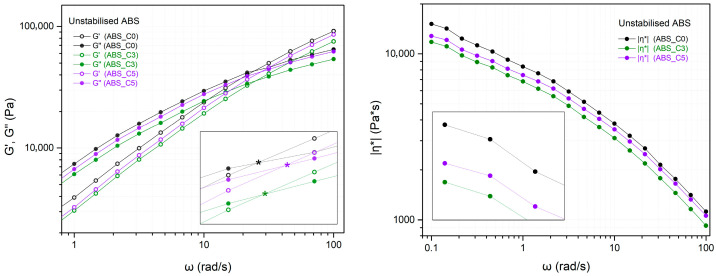
The storage modulus (*G*′), loss modulus (*G*″), and complex viscosity (η*) of unstabilized ABS in C0 vs. the unstabilized ABS after 3 and 5 reprocessing cycles at 250 °C, in frequency sweep in 0.1–100 rad/s at 220 °C. Inset in the G′-G″ plots show a magnified view of the crossover region (*G*′ = *G*″), where the crossover point is marked with an asterisk (*).

**Figure 4:**
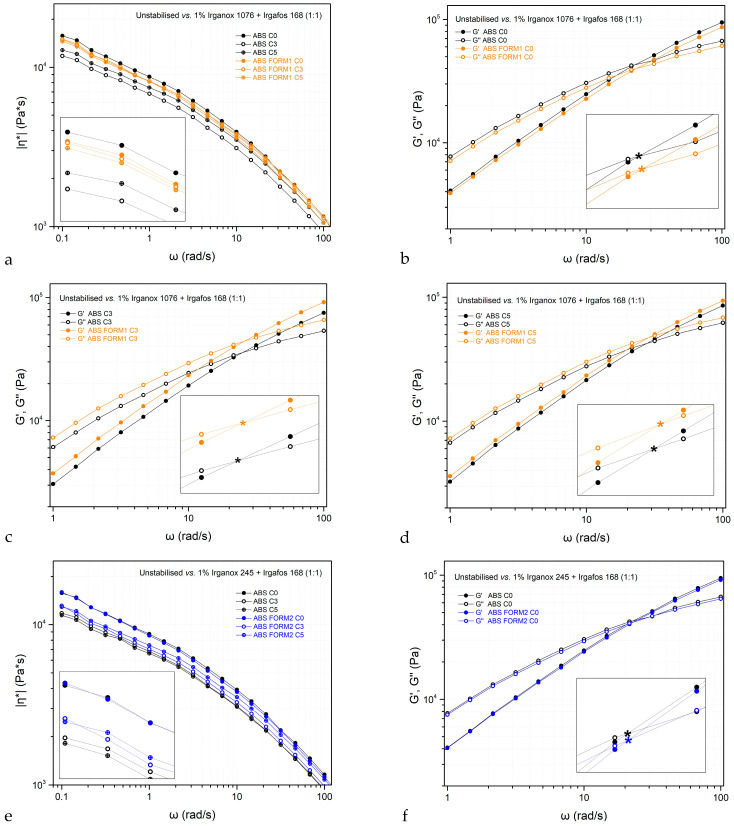

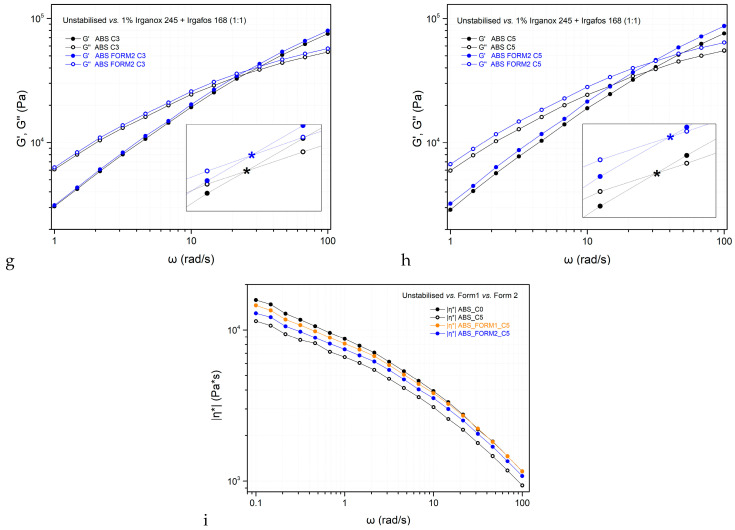
The storage modulus (*G*′), loss modulus (*G*″), and complex viscosity (η*) of unstabilized ABS vs. stabilized ABS with F1 and F2 in reprocessing cycles 0, 3 and 5 at 250 °C, in 0.1–600 rad/s at 220 °C. Graphs (**a**,**e**) show the evolution of complex viscosity (η*) curves of ABS with F1 and F2 respectively throughout reprocessing cycles. Graphs (**b**–**g**) show the the *G*′-*G*″ plots of ABS with F1 and F2 respectively for different reprocessing cyles; (**h**) insets magnified views of the crossovers regions (*G*′= *G*″), where the crossover point is marked with asterisks (*). Graph (**i**) compares the complex viscosity (η*) of unstabilized ABS with stabilized ABS with F1 and F2 in reprocessing cycles 0 and 5.

The dynamic rheological behaviour of the initial and reprocessed ABS, as well as the two reprocessed stabilized compounds at cycles 0 and 5, was studied by oscillatory frequency sweep tests at 0.1–100 rad/s measured at 220 °C within the linear viscoelastic region (LVE, 0.05% strain). The respective storage (*G*′) and loss (*G*″) moduli and complex viscosity (η*) are presented as a function of angular frequency (*ω*), and are presented in Figure 3 and Figure 4, as well as in Table 3.

In dynamic oscillatory shear tests, as the angular frequency increases, the complex viscosities of all samples decreased because thermoplastic polymers generally show shear-thinning behaviour with increasing shear rate [46]. The most direct and significant indicator of a polymer’s stability during reprocessing is the change in its complex viscosity at a low frequency (0.1 rad/s), which is approx. zero-shear viscosity (*η*_0_). Zero-shear viscosity is highly related to changes in the material’s molecular weight, and its substantial reduction is a clear indication of chain scission [47,48]. For Newtonian fluids, *η*_0_ is usually taken at the plateau of η* (η* = *η*′ − i∙*η*″) in the low frequency terminal region; however, decisive conclusions for the melt strength can be drawn by juxtaposing the η* values taken at low *ω* (i.e., 0.1 rad/s. In initial neat ABS, *η*_0_ was reduced in the 5th cycle by 27.5% and stabilized with formulation 2 by 19.3%, providing evidence of deterioration of rheological properties driven by chain scission. In contrast, formulation 1 exhibits even better stability with a slight reduction of only 3.3% in this key rheological parameter. It is worth noting that the complex viscosity of the neat ABS at the third reprocessing cycle is lower than that observed at the fifth cycle. This behaviour indicates that, although chain scission is the dominant mechanism during the initial stages of reprocessing, additional reactions may occur at higher processing histories. In particular, the slight increase in viscosity at later cycles suggests competing degradation mechanisms, such as limited branching or recombination reactions, potentially associated with the polybutadiene phase under thermo-oxidative conditions.

Furthermore, the complex viscosity in the high-frequency region (100 rad/s) or high-shear viscosity provides information related to the material processability behaviour during industrial processes, such as extrusion and injection moulding. In this case, similarly, the initial neat ABS material presented a substantial reduction in the fifth cycle (19.2%), while formulation 2 showed good stability with a slight reduction equal to 3.6%, which is also in accordance with the obtained MFR measurements. In the case of formulation 1, the high-shear viscosity is slightly increased (9.7%) in the fifth cycle. This behaviour is probably attributed to branching or cross-linking reactions.

Another value from which valuable information can be found is the changes in the cross-over frequency (*ω*_c_) and cross-over modulus (*G*_c_). The cross-over frequency shifts are related to the polymer average relaxation time and therefore the average molecular weight, while the changes in the cross-over moduli are related to the distribution of the molecular weight and hence polydispersity [49].

In this case, the cross-over angular frequency shifts observed were in accordance; a shift in crossover frequency to a higher frequency meant a reduction in the average relaxation time, which means a decrease in average molecular weight. Starting with the evaluation of ω_c_, in the fifth cycle, it shifted to higher values in all three cases by 21.1% (neat ABS), 21.7% (FORM1), and 27.9% (FORM2), indicating faster relaxation times and a reduction in average molecular weight. However, the changes in the cross-over moduli (*G_c_*) reveal differences in the degradation mechanisms. Neat ABS showed a decrease in *G_c_* by 12.9% in the fifth cycle, meaning broadening in the molecular weight distribution, which is expected for the random chain scission degradation mechanism, creating a heterogeneous mix of molecular weights (chain lengths). In the case of restabilised formulations, it was not the case; in formulation 1, the *G_c_* was increased by 18.0% and in the case of formulation 2 by 5.7% in the fifth cycle. This behaviour suggests a narrower molecular weight distribution. Higher *ω*_c_ combined with a higher *G*_c_ implies a more selective degradation mechanism. An explanation is that the chain scission occurs to the high molecular weight tail of the distribution, decreasing the average molecular weight, while narrowing the distribution. Another possible mechanism that could explain the narrowing in the molecular weight distribution is seen through the crosslinking of the smaller chain lengths first, increasing their molecular weight closer to those of higher molecular weight, narrowing the distribution [50].

#### 3.1.3. Molecular Weight Distribution

**Figure 5:**
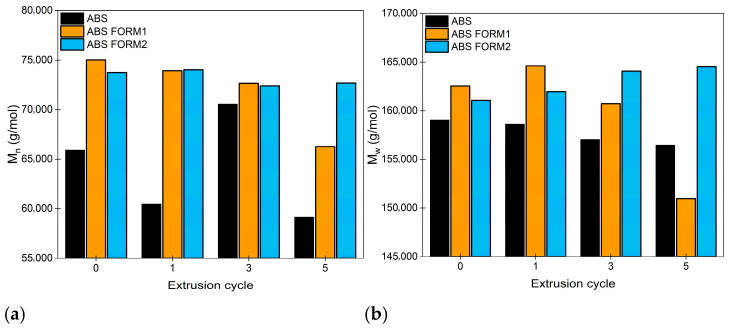
(**a**) $\bar{M_{n}}$ and (**b**) $\bar{M_{w}}$ of neat ABS, ABS FORM1 and ABS FORM2 as functions of reprocessing cycles.

GPC was performed to further investigate the effect of thermomechanical degradation throughout reprocessing cycles. Both number-average ($\bar{M_{n}}$) and weight-average ($\bar{M_{w}}$) molecular weights were determined for reprocessing cycles 1, 3, and 5, and are presented in Figure 5. Starting with the neat ABS, ($\bar{M_{n}}$) decreased from 66,000 to 59,000 g/mol (10% decrease) while $\bar{M_{w}}$ remained relatively stable at the 156,000–159,000 g/mol range, meaning only 1.6% decrease along the reprocessing cycles. This behaviour pointed out to chain scission as the predominant degradation mechanism (rather than crosslinking), leading to smaller, low-MW chains [51]. In contrast, the ABS_FORM1 and ABS_FORM2 materials started from higher initial $\bar{M_{n}}$ (~75,000 vs. 66,000 g/mol for the neat) and $\bar{M_{w}}$ (~163,000 vs. 159,000 g/mol for the neat) values and better maintained them with multiple extrusions, especially ABS_FORM2. Indeed, $\bar{M_{n}}$ decreased from 75,000 to 66,000 g/mol (10% decrease) for ABS_FORM1, while ABS_FORM2 better maintained its values, i.e., from 74,000 to 73,000 g/mol (only 1% decrease). Additionally, $\bar{M_{w}}$ slightly reduced from 151,000 to 162,000 g/mol range (7.1% decrease) for ABS_FORM1, while for ABS_FORM2 $\bar{M_{w}}$ slightly increased from 161,000 to 164,000 g/mol range (2.1% increase). For all materials, polydispersity index (*Ð*) did not significantly vary, namely from 2.2 to 2.6.

These results are in accordance with the dynamic rheological characteristics analyzed above; an increase in cross-over angular frequency (*ω*_c_) and cross-over modulus (*G*_c_) throughout reprocessing cycles indicates faster relaxation times and a stiffer network at the crossover point. Together, these changes show that reprocessing degrades the PB phase and affects the SAN matrix, reducing viscosity and broadening molecular weight distribution. Thus, zero-shear viscosity decrease correlates with *M*_n_ reduction and increased *ω*_c_ and *G*_c_ reflect changes in polymer chain mobility and network structure due to reprocessing-induced degradation [52,53].

#### 3.1.4. Thermal Properties

**Figure 6:**
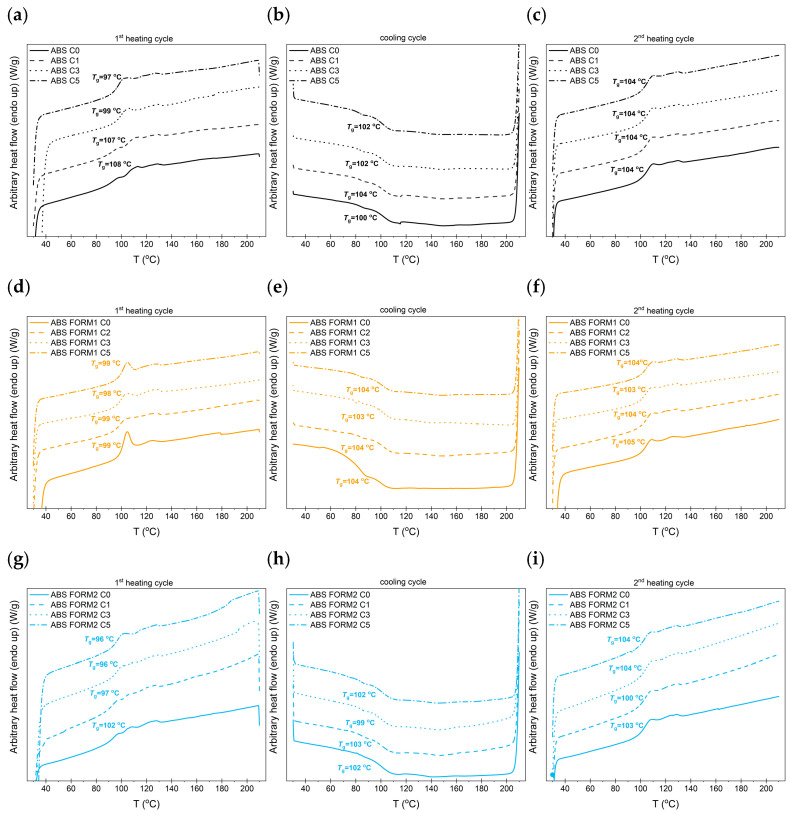
DSC curves of the first heating, cooling and second heating cycle of (**a**–**c**) neat ABS, (**d**–**f**) ABS_FORM1, and (**e**–**i**) ABS_FORM2, as functions of reprocessing cycles.

Turning the focus on thermal properties, DSC thermograms were studied (Figure 6), where the results of the first heating cycle were initially assessed to showcase any alterations directly after reprocessing. The *T*_g_s of all materials were around 96–108 °C associated with the styrenic component, while around 127–131 °C, there was an endotherm peak associated with reordering/relaxation movements after glass transition [39,54]. Starting with the neat ABS (Figure 6a), *T*_g_ decreased from 108 to 97 °C with increasing reprocessing cycles, evidence of the chain scission occurring along its backbone, as was also indicated from the rheological and GPC analysis. In contrast, ABS_FORM1 kept its *T*_g_ at 98–99 °C (Figure 6d) along the reprocessing cycles, while ABS_FORM2 was at 97–109 °C (Figure 6g). For both formulations, the *T*_g_s of the initial compounded materials (cycle 0) were slightly lower than that of the neat one, possibly due to the influence of the incorporated additives on the macromolecular structure. Evaluating the data from cooling and second heating data to erase any thermal annealing effects and/or history, no significant variations were revealed, and the values lay in the same vicinity, i.e., for neat ABS at 100–104 °C, for ABS_FORM1 at 103–105 °C, and ABS_FORM2 in the 100–104 °C range.

**Figure 7:**
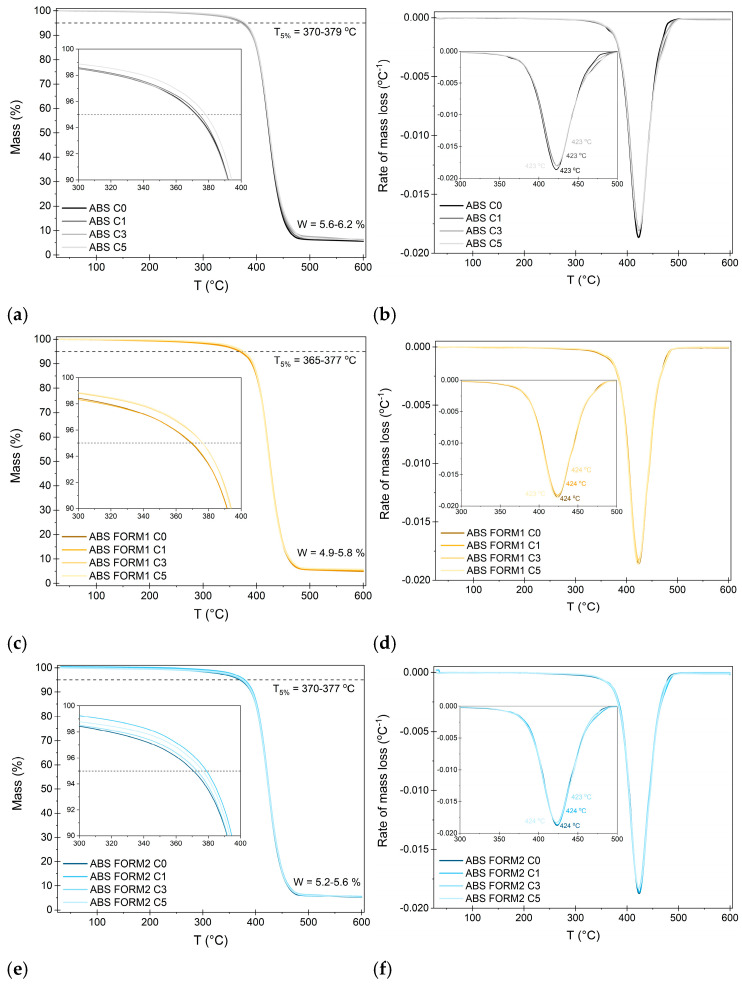
TGA curves and first derivatives of (**a**,**b**) neat ABS, (**c**,**d**) ABS_FORM1, and (**e**,**f**) ABS_FORM2, as functions of reprocessing cycles.

Regarding thermal stability, no significant mass loss was observed until *T*_5%_, which varied from 365 to 379 for all materials, with significant variations neither between materials nor reprocessing cycles (Figure 7). Thermal decomposition occurred in a single step, peaking at around 423–424 °C. In all cases, the residues at 600 °C were around 4.9–6.2%.

#### 3.1.5. ATR-FTIR Results

Changes in the macromolecular structure during reprocessing were also observed in the relevant ATR-FTIR spectra. Starting with the identification of ABS characteristic peaks, in the mid-wavenumber region (MWIR), the C−H stretching vibrations of the aromatic ring (at 3080, 3060, 3025 and 3000 cm^−1^) as well as the aliphatic ones (2920 and 2850 cm^−1^) were clearly seen, due to the presence of both the styrene and polybutadiene phase, while the C≡N stretching vibration at 2237 cm^−1^ was attributed to acrylonitrile phase. In the low-wavenumber region (LWIR), the 1494 cm^−1^ (stretching vibration of C−H of the ABS aromatic ring) and 1451 cm^−1^ (scissoring mode of −CH_2_ present in ABS) were evident. Also, in LWIR, the aromatic C−H and =CH wagging and scissoring peaks of ABS were located at 759 and 698 cm^−1^. Also, the PB phase was easily detectable at 965 cm^−1^ (=C−H deformation of PB *trans*-1,4) and 910 cm^−1^ (=C−H out-of-plane deformation of PB *vinyl*-1,2).

**Figure 8:**
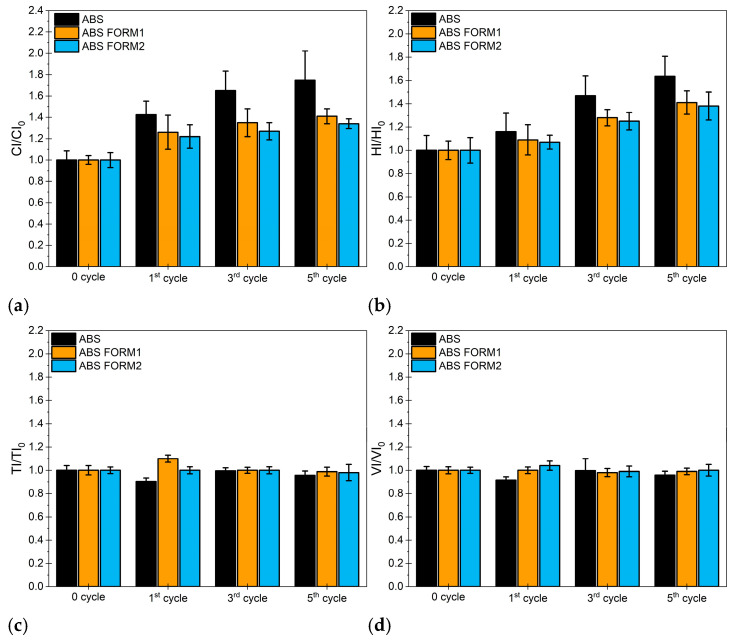
Calculated indices, (**a**) CI/CI_0_, (**b**) HI/HI_0_, (**c**) TI/TI_0_, and (**d**) VI/VI_0_, of neat ABS, ABS_FORM1, and ABS_FORM2, as functions of reprocessing cycles.

Changes due to thermomechanical degradation involve a potential increase in the hydroxyl group region at 3200–3650 cm^−1^, carbonyl group at 1680–1850 cm^−1^, as well as *trans*-1,4 (at 930–990 cm^−1^) and *vinyl*-1,2 (at 890–930 cm^−1^) group generations. The pertinent regions were integrated and normalized to the styrenic peak of the benzene ring located at 1494 cm^−1^ (integration region at 475–1510 cm^−1^), which remained relatively constant. Then, each index was divided into its initial value, e.g., CI/CI_0_, to provide a clear picture of the evolution of degradation products along the reprocessing cycles (Figure 8). Indeed, the neat ABS demonstrated a greater, progressive increase in the carbonyl and hydroxyl groups region (Figure 8a,b), evidence of more pronounced degradation, while the ABS FORM1 and especially ABS FORM2 materials, which were more efficiently stabilized, demonstrated lower indices overall. A minor decrease was observed in the *trans*-1,4 and *vinyl*-1,2 for the neat ABS along the reprocessing cycles, a possible indication of crosslinking or branching.

By correlating the results from MFR, rheology, GPC and FTIR analyses, a more comprehensive interpretation of the degradation behaviour can be obtained. The progressive increase in MFR observed during the initial reprocessing cycles is consistent with the reduction in molecular weight determined by GPC, confirming that chain scission is the dominant mechanism. This is further supported by rheological measurements, which show a decrease in viscosity and changes in viscoelastic response, indicating reduced entanglement density and enhanced chain mobility. At higher reprocessing cycles, deviations from this trend in the neat ABS suggest the presence of competing degradation mechanisms. While chain scission remains predominant, the decrease in MFR in the fifth cycle, as well as an increase in zero-shear viscosity, may indicate the formation of branched or weakly crosslinked structures. This interpretation is also supported by the broadening of the molecular weight distribution observed in GPC, as well as the continued evolution of oxidation-related functional groups detected by FTIR.

Overall, the combined analysis performed indicates that thermomechanical degradation of the recycled ABS proceeds through a balance of chain scission and secondary recombination reactions, particularly involving the polybutadiene phase.

#### 3.1.6. Colour Properties

**Figure 9:**
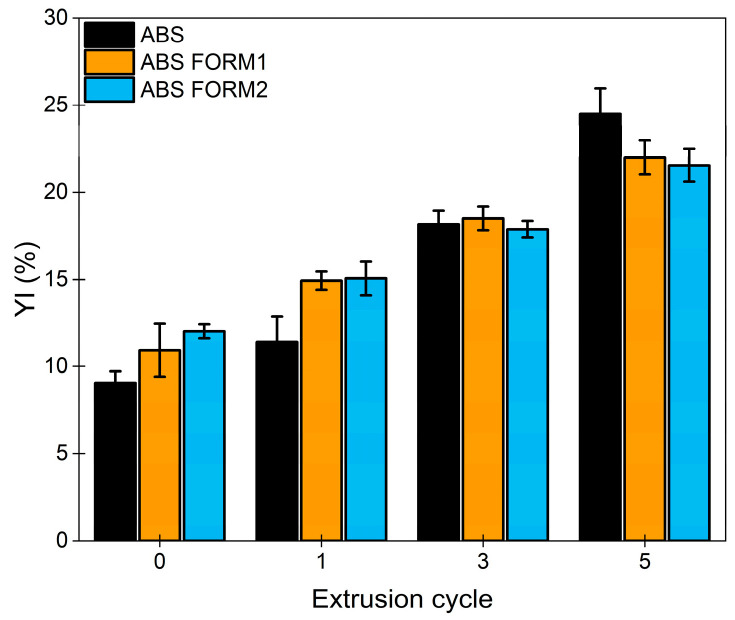
Yellowness Indexes (YI) of neat ABS, ABS_FORM1 and ABS_FORM2, as functions of reprocessing cycles.

The colour stability of the recycled ABS (neat and stabilized) during reprocessing was evaluated using the Yellowness Index (YI), which is commonly used as a sensitive indicator of thermo-oxidative degradation in polymers. As shown in Figure 9, YI increased progressively with the number of reprocessing cycles for the non-stabilized material up to 25%, indicating the formation of yellow chromophore species. In contrast, the stabilized materials exhibited slightly lower YI values (picking at ~20%) and a slower rate of yellowing, demonstrating improved resistance to oxidation. It should be noted that the stabilized samples exhibited a slightly higher initial Yellowness Index (~12%) compared to the neat material (~9%), which can be attributed to the incorporation and processing of antioxidant additives. As a result, direct comparison of absolute YI values after multiple reprocessing cycles may not fully reflect the effectiveness of the stabilization system. Instead, the evolution of YI during reprocessing provides a more meaningful assessment of thermo-oxidative degradation. In this context, the stabilized samples show a reduced increase in YI with increasing number of cycles, indicating that the antioxidant system effectively suppresses the formation of additional chromophoric oxidation products.

Although the reduction in YI appears relatively modest, this result is relevant considering that mitigating yellowing in WEEE-derived ABS is inherently challenging. These materials originate from heterogeneous waste streams with varying compositions, service lifetimes, and prior thermal and environmental ageing histories, all of which contribute to pre-existing and non-uniform discoloration.

This behaviour is consistent with the ATR-FTIR results, which showed reduced formation of carbonyl-containing degradation products in the stabilized samples. The relatively low standard deviation across the ten measured points confirms the uniformity of the films and the reliability of the measurements. Overall, the YI results confirm that antioxidant addition effectively mitigates colour degradation during mechanical recycling of ABS.

#### 3.1.7. Tensile Properties

**Figure 10:**
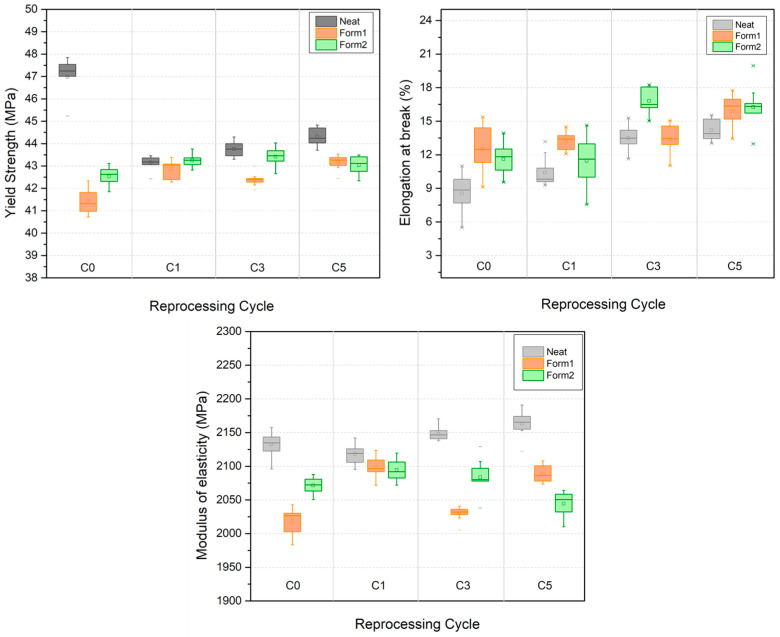
Comparison of tensile strength, Young’s modulus, and strain at break of neat ABS and the two compounds with the antioxidants at reprocessing cycles 0, 1, 3, and 5.

The tensile properties yield strength (*σ*, MPa), modulus of elasticity (*E*, MPa) and strain at break (*ε*, %) of the ABS throughout the five reprocessing cycles are presented in Table 4 and Figure 10. The neat material exhibited an initial yield strength of 47 MPa, which decreased after the first reprocessing cycle to approximately 43 MPa and then remained relatively stable up to five cycles. A similar trend was observed for the antioxidant-containing formulations, with yield strength values consistently in the range of 42–43 MPa, indicating that multiple reprocessing cycles do not significantly compromise the load-bearing capacity of the material. The elastic modulus remained essentially constant for all materials and processing conditions, with values between 2000 and 2150 MPa, suggesting that the stiffness of the SAN matrix and rubber phase morphology were not significantly affected by thermomechanical degradation.

In contrast, the strain at break showed a clear increasing trend with reprocessing cycles. The neat ABS exhibited an increase from 8.54% at cycle 0 to 14.20% at cycle 5, while the stabilized formulations reached values up to 16–17%. This increase in ductility can be attributed to a combination of degradation-induced structural alterations in the polymer backbones. Chain scission during reprocessing led to a reduction in molecular weight, according to GPC results, as well as an increase in the entanglement density, facilitating increased chain mobility and promoting more ductile behaviour. In addition, the mechanical response of ABS is strongly influenced by the PB phase; degradation-induced structural modifications in the PB phase, as was indicated by ATR-FTIR analysis, affect its elasticity and its interaction with the SAN matrix, and, therefore, increase the energy absorption during deformation. Furthermore, the formation of low-molecular-weight species during degradation may contribute to a plasticisation-like effect, which further increases ductility [55]. The slightly higher elongation observed for the stabilized formulations suggests that antioxidant addition helps maintain a favourable balance between chain scission and structural integrity, preventing excessive degradation while preserving ductility.

### 3.2. Production of Filaments for 3D Printing

**Figure 11:**
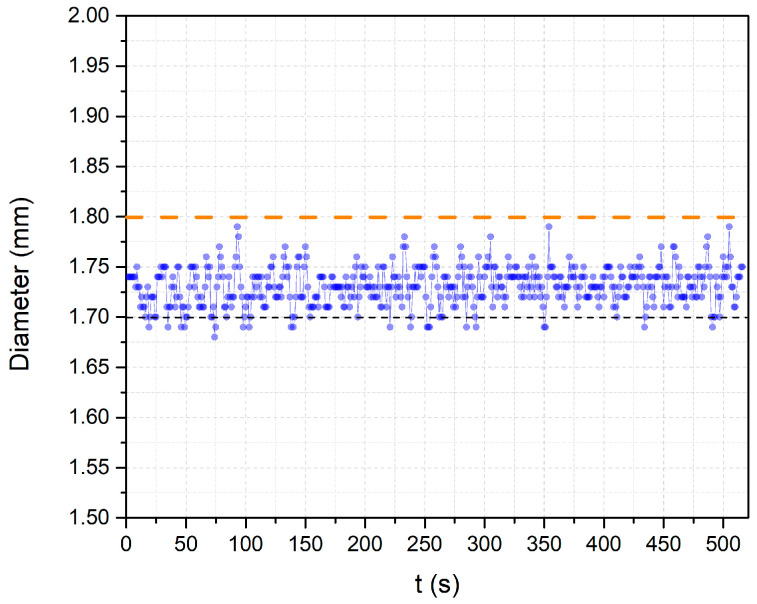
Monitoring of filament diameter. The lower limit of 1.70 mm diameter is indicated by a black dotted line, while the upper limit is indicated by an orange dotted line.

Based on the thermomechanical degradation results, the second formulation (1% Irganox^®^ 245 + Irgafos^®^ 168 (1:1)) was selected for filament production due to its superior retention of melt flow characteristics and mechanical properties. The selected recycled ABS was granulated, dried at 80 °C for 4 h, and processed using a single-screw extruder (Boston Matthews N3025, Boston Matthews, Worcester, UK) equipped with a 2 mm circular die. The extrusion temperature profile ranged from 180 to 210 °C, with a screw speed of 10 rpm. The extrudate was water-cooled in two consecutive baths (first at 50 °C, followed by room temperature) and collected using a controlled winding system. The dimensional stability of the produced filament was assessed via continuous diameter monitoring. The filament exhibited an average diameter of 1.75 ± 0.05 mm, remaining within the acceptable tolerance range for fused filament fabrication processes (±0.05 mm). Furthermore, the filaments exhibited low ovality, indicating a near-circular cross-section and uniform material flow at the die exit. This behaviour, as well as the low diameter fluctuation, reflects stable viscoelastic properties and sufficient melt strength of the selected recycled ABS formulation, which are essential for consistent feeding during fused filament fabrication. The diameter was monitored using a three-axis laser before the spooling. The diameter monitoring is presented in Figure 11.

In addition, no significant flow instabilities or filament breakage were observed during extrusion, indicating stable processing behaviour. The combination of dimensional consistency, low ovality, and stable extrusion suggests that the material meets the fundamental requirements for reliable filament handling and feeding in FFF systems, supporting its suitability for additive manufacturing applications.

## 4. Conclusions

This study evaluated the thermomechanical degradation behaviour of post-consumer ABS recovered from sorted WEEE streams and assessed the effectiveness of antioxidant stabilization during multiple reprocessing cycles. The results demonstrate that repeated melt processing induces moderate molecular degradation, reflected in a reduction in melt flow rate, intrinsic viscosity and progressive increase in the carbonyl indexes as well as changes in colour, while the modulus of elasticity remained largely unaffected. At the same time, an increase in strain at break was observed, indicating enhanced chain mobility due to chain scission. The incorporation of antioxidant stabilizers proved to be effective in mitigating thermo-oxidative degradation. Stabilized formulations exhibited improved retention of rheological and mechanical properties while reducing the yellowing compared to the non-stabilized material. These findings confirm that appropriate stabilization strategies can preserve the functional performance of recycled ABS even after multiple processing cycles. Importantly, the chosen stabilized formulations demonstrated adequate melt strength and rheological stability for filament extrusion. The produced filaments exhibited consistent diameter and low ovality within the tolerance required for fused filament fabrication, confirming their suitability as feedstock for additive manufacturing. Overall, the results highlight that post-consumer ABS from WEEE streams can be effectively valorised through mechanical recycling when combined with appropriate stabilization strategies. The successful production of dimensionally stable filaments demonstrates the potential of these materials for high-value applications such as additive manufacturing, contributing to the development of circular material flows for styrenic plastics.

## Figures and Tables

**Table 1:** Process parameters for 10.0 wt.%. ABS masterbatches of primary and secondary antioxidants (1:1), as well as for the final compounds with 1% wt. additives.

Materials	T_melt_ (°C)	Z8 (°C)	Z7 (°C)	Z6 (°C)	Z5 (°C)	Z4 (°C)	Z3 (°C)	Z2 (°C)	Z1 (°C)	Screw Speed (rpm)	Vacuum
ABS_MB (a)	210	-	-	210	215	210	200	195	195	80	Yes
ABS_MB (b)	200	-	-	210	205	200	200	195	195	80	Yes
ABS_FORM1_C0	210	215	215	212	210	210	205	200	190	130	No
ABS_FORM1_C0	210	215	212	215	212	212	210	205	190	130	No

**Table 2:** Nomenclatures of all the samples.

Samples	Nomenclature
ABS waste	ABS_C0
ABS waste after one reprocessing cycle	ABS_C1
ABS waste after three reprocessing cycles	ABS_C3
ABS waste after five reprocessing cycles	ABS_C5
ABS masterbatch (a) consisted of 10% Irganox^®^ 1076 + Irgafos^®^ 168 (1:1)	ABS_MB (a)
ABS masterbatch (b) consisted of 10% Irganox^®^ 245 + Irgafos^®^ 168 (1:1)	ABS_MB (b)
ABS waste stabilized with 1% Irganox^®^ 1076 + Irgafos^®^ 168 (1:1) (FORM1)	ABS_FORM1_C0
ABS waste stabilized with 1% Irganox^®^ 1076 + Irgafos^®^ 168 (1:1) (FORM1) after one reprocessing cycle	ABS_FORM1_C1
ABS waste stabilized with 1% Irganox^®^ 1076 + Irgafos^®^ 168 (1:1) (FORM1) after three reprocessing cycles	ABS_FORM1_C3
ABS waste stabilized with 1% Irganox^®^ 1076 + Irgafos^®^ 168 (1:1) (FORM1) after five reprocessing cycles	ABS_FORM1_C5
ABS waste stabilized with 1% Irganox^®^ 245 + Irgafos^®^ 168 (1:1) (FORM2)	ABS_FORM2_C0
ABS waste stabilized with 1% Irganox^®^ 245 + Irgafos^®^ 168 (1:1) (FORM2) after one reprocessing cycle	ABS_FORM2_C1
ABS waste stabilized with 1% Irganox^®^ 245 + Irgafos^®^ 168 (1:1) (FORM2) after three reprocessing cycles	ABS_FORM2_C3
ABS waste stabilized with 1% Irganox^®^ 245 + Irgafos^®^ 168 (1:1) (FORM2) after five reprocessing cycles	ABS_FORM2_C5

**Table 3:** G′/G″ cross-over at the respective angular frequency and complex viscosity at low and high frequency of the different unstabilized and stabilized ABS.

Sample	Moduli Cross-Over (*G_c_*) [Pa]	Frequency Cross-Over (*ω*_c_) [rad/s]	Zero-Shear Complex Viscosity (η* at 0.1 rad/s) [Pas]	High-Shear Complex Viscosity (η* at 100 rad/s) [Pas]
ABS_C0	42,887	22.83	15,760	1158
ABS_C3	35,227	24.69	11,783	925
ABS_C5	37,348	27.64	11,430	936
ABS_FORM1_C0	39,988	23.38	15,023	1059
ABS_FORM1_C3	42,396	24.34	14,918	1131
ABS_FORM1_C5	47,173	28.45	14,523	1162
ABS_FORM2_C0	41,841	22.90	15,953	1120
ABS_FORM2_C3	37,237	24.22	13,108	981
ABS_FORM2_C5	44,216	29.30	12,870	1080

**Table 4:** Tensile testing results of neat ABS and two compounds for reprocessing cycles 0, 1, 3, and 5.

Sample	Yield Strength (MPa)		Modulus of Elasticity (MPa)		Strain at Break (%)	
	Mean	SD	Mean	SD	Mean	SD
ABS_C0	47.0	0.86	2132	17	8.5	1.9
ABS_FORM1_C0	41.4	0.55	2018	21	12.5	2.1
ABS_FORM2_C0	42.5	0.42	2071	12	11.6	1.3
ABS_C1	43.2	0.30	2118	15	10.4	1.3
ABS_FORM1_C1	42.9	0.40	2098	17	13.2	0.8
ABS_FORM2_C1	43.3	0.26	2094	16	11.5	2.0
ABS_C3	43.8	0.35	2148	10	13.5	1.1
ABS_FORM1_C3	42.4	0.27	2030	10	16.8	1.1
ABS_FORM2_C3	43.4	0.42	2082	26	13.4	1.4
ABS_C5	44.3	0.37	2163	19	14.2	0.9
ABS_FORM1_C5	43.2	0.32	2088	13	15.9	1.4
ABS_FORM2_C5	43.0	0.40	2044	19	16.3	1.8

## Data Availability

The data presented in this study are available on request from the corresponding authors due to data protection and confidentiality requirements within a European Union-funded project.
